# Utilization of telemedicine to support caregivers of young children with ASD and their Part C service providers: a comparison of intervention outcomes across three models of service delivery

**DOI:** 10.1186/s11689-021-09387-w

**Published:** 2021-09-15

**Authors:** Laura L. Corona, J. Alacia Stainbrook, Kathleen Simcoe, Liliana Wagner, Bethena Fowler, Amy S. Weitlauf, A. Pablo Juárez, Zachary Warren

**Affiliations:** 1 Vanderbilt Kennedy Center/Treatment and Research Institute for Autism Spectrum Disorders, Vanderbilt University Medical Center, 1207 17th Avenue South, Nashville, TN 37203 USA; 2 Department of Pediatrics, Vanderbilt University Medical Center, Nashville, TN USA; 3 Department of Psychiatry and Behavioral Sciences, Vanderbilt University Medical Center, Nashville, TN USA; 4 Department of Special Education, Vanderbilt University, Nashville, TN USA

## Abstract

**Background:**

Families of young children with autism spectrum disorder (ASD) frequently experience barriers to accessing evidence-based early intervention services. Telemedicine presents an opportunity to increase access to these services, particularly for families in rural and under-resourced areas. The present article describes a brief behavioral intervention and support model for families of young children with concerns for ASD. In the context of the COVID-19 pandemic, this service model shifted to telemedicine-only service delivery, resulting in an opportunity to analyze intervention outcomes from services delivered either via traditional in-person visits, telemedicine-only sessions, or a hybrid model including both in-person and telemedicine sessions.

**Methods:**

Data are presented for 115 families with toddlers 16-33 months of age who participated in a six-session behavioral intervention and support service model either in-person, through telemedicine, or through a hybrid service model. This intervention was available for families referred for ASD evaluation through the state Part C early intervention program. Intervention feasibility, fidelity of implementation, child outcomes, and stakeholder satisfaction are compared across service delivery models.

**Results:**

Caregivers, behavioral consultants, and Part C early intervention providers reported satisfaction with services, regardless of service delivery model. Caregivers and consultants also reported positive child outcomes. Statistically significant differences emerged for caregiver- and consultant-reported child outcomes in some domains, with stakeholders in the telemedicine-only group reporting slightly less improvement, compared to stakeholders in the in-person-only group. Caregivers and consultants in the telemedicine-only group also provided qualitative feedback on benefits and challenges related to telemedicine services.

**Conclusions:**

Both caregivers and behavioral consultants reported positive outcomes following a brief behavioral intervention and support model targeted at families of young children with concern for ASD. Stakeholders reported improvement in child behavior and satisfaction with services across in-person, telemedicine-only, and hybrid models of service delivery. These results suggest that telemedicine presents a promising opportunity for increasing service access. Additional research is needed to continue optimizing the experience of telemedicine-based service delivery for both families and intervention providers.

## Background

Together, the increasing prevalence of autism spectrum disorder (ASD) [1] and an emphasis on early identification of ASD during the toddler years [2, 3] have resulted in a rising number of young children in need of autism-focused diagnostic and early intervention services. Early, targeted intervention is reliably linked to positive outcomes for children with ASD [4, 5]. However, numerous barriers impact families’ access to early diagnostic and intervention services [6–8]. Specifically, the therapies recommended for most children at the time of ASD diagnosis (i.e., applied behavior analysis (ABA) provided under the direction of a board certified and licensed behavior analyst) are frequently difficult to obtain, particularly for families who live in under-resourced communities [9, 10]. Addressing these barriers requires creative approaches that leverage both existing service systems and novel tools for reaching families [11].

Given the challenges accessing intervention services, many children diagnosed with ASD during toddlerhood rely on their state Part C early intervention (EI) systems for this care. Part C systems refer to federal grant programs that assist states in operating statewide EI services for children with disabilities under 3 years of age and their families [12]. However, state EI systems vary significantly regarding their abilities to offer empirically validated treatments that specifically target ASD symptoms [13]. Some can integrate treatments such as ABA as part of standard care, whereas others offer broader developmental services (such as speech and occupational therapy) that may or may not be tailored to children on the autism spectrum. Access to these services and expertise are even more challenging in rural areas where providers may be limited in availability and themselves have limited access to professional development and support opportunities related to ASD and evidence-based practices [14, 15].

Telehealth represents a promising avenue for increasing access to services for individuals with ASD [6, 16]. For example, research comparing outcomes between groups of caregivers taught to conduct functional analyses and functional communication training (a) in-home with a clinician, (b) clinic-based with the clinician available via telehealth, and (c) home-based with the clinician available via telehealth, yielded comparable reductions in problem behavior [17, 18]. Telemedicine-based approaches have also been used to coach caregivers of young children through implementation of intervention strategies. Ingersoll et al. found positive outcomes related to caregiver implementation fidelity, caregiver perceptions of child, and child language and social skills outcomes when caregivers were provided with a therapist-assisted, telehealth-based, parent-mediated intervention for young children with ASD [19]. Vismara et al. found positive outcomes related to caregiver implementation of the Early Start Denver Model and satisfaction with intervention support when caregivers were provided with parent coaching via telehealth [20]. Telemedicine has also been used in the context of ASD assessment for young children and has demonstrated caregiver and clinician acceptability [17, 18], as well as measurable effects on referrals to tertiary care assessment settings [19].

Over the past months, the COVID-19 pandemic has accelerated providers’ adoption of telehealth modalities [20, 21]. As in-person visits were suspended or reduced, telehealth-based appointments presented an opportunity to continue meeting families’ needs as wait lists for diagnostic appointments and EI services continued to grow. This unprecedented service system shift, while extremely challenging for families and service systems, has provided opportunities to pilot novel service delivery modalities and collect and analyze data on the impact of transitioning from in-person or hybrid service delivery models to fully telemedicine-based models of care.

Our university medical center has a lengthy and collaborative partnership with the state Part C system. Prior to the COVID-19 pandemic, this partnership had already facilitated the development of a novel early ASD identification and intervention model inclusive of telemedicine options for families in rural and under-resourced communities [18, 19]. The model is designed to increase families’ access to care, as well as to build capacity of Part C service providers, through the provision of diagnostic consultation and subsequent behavioral intervention and support services using both in-person and telemedicine-based service delivery options. In response to COVID-19, our university medical center continued to partner closely with the state Part C program, shifting to a telemedicine-only model of service provision to ensure that families could access both diagnostic and intervention services despite the inability to travel to specialty clinics or send behavioral consultants into family homes.

In this work, we describe the transition to a telemedicine-only service delivery model in response to the COVID-19 pandemic, with a particular focus on the delivery of EI services. Intervention feasibility, fidelity of implementation, child outcomes, and stakeholder satisfaction are compared across service delivery models (i.e., in-person, hybrid, telemedicine).

## Methods

### Overview of service model

In partnership with our state Part C system, our university-affiliated medical center developed a model of service delivery inclusive of comprehensive diagnostic evaluations and subsequent behavioral intervention and support services for children who were referred for ASD evaluation. This model is funded through a grant from the state Part C EI system and serves counties within four pre-identified regions of the state.

Within this model, children were eligible if they were receiving Part C EI services, had a developmental therapist, and were under 33 months of age, to allow time for service completion prior to the child reaching 3 years of age and exiting the Part C program. Participating families were referred for ASD evaluation by the family’s Part C service coordinator. Eligible children first participated in a diagnostic evaluation with a licensed clinical provider. Regardless of the outcome of the diagnostic evaluation (ASD vs. no ASD/other diagnosis), children were then eligible for six sessions of behavioral intervention and support services. The majority of participants had a diagnosis of ASD (78%). The remaining participants held a caregiver-reported diagnosis of developmental delay (13%) or were marked as having “Other” diagnoses (9%; e.g., Chromosome 8, Monosomy 8p; septo-optic dysplasia; mosaic Down syndrome). Behavioral intervention and support services were discussed with the family immediately after the diagnostic evaluation. Offered services focused on (1) providing caregivers with immediate support following evaluation for ASD, (2) teaching caregivers how to embed evidence-based strategies for children with ASD and related developmental concerns into their daily routines, and (3) partnering with and supporting providers within the EI system in helping family members to maintain and generalize use of these strategies.

Prior to the COVID-19 pandemic, this service model included embedded options for telemedicine-based service delivery for families in geographically isolated regions or for whom travel represented a significant barrier. Though services were primarily delivered through traditional, in-person visits, this model piloted novel approaches to telemedicine-based ASD assessment [18, 19], as well as opportunities for hybrid intervention services (i.e., a mix of in-person and telemedicine visits).

At the onset of the COVID-19 pandemic, in the context of social distancing mandates and the suspension of in-person services, our team transitioned both diagnostic and intervention services exclusively to telemedicine modalities. Families already participating in intervention services in traditional in-person format transitioned to telemedicine services. Families initiating services during the COVID-19 pandemic received telemedicine services only. Analysis of outcomes of tele-assessment services has been published elsewhere [22]. In what follows, we describe our intervention service model and report implementation and outcomes across intervention models (i.e., in-person, telemedicine-only, and hybrid service delivery).

### Description of intervention

Participating families were eligible to receive six intervention sessions driven by one of five topic-specific, manualized curriculum modules developed by a team of in-house behavior analysts, speech-language pathologists, and psychologists. Module curriculum topics included the following: Addressing challenging behavior, basics of communication, developing social and play skills, beginning toilet training, and addressing sleep concerns (see Table 1)*.* The curriculum modules applied evidence-based strategies, based on the strategies included in the 2020 National Clearinghouse on Autism Evidence and Practice [23], such as prompting, differential reinforcement, and visual supports.

**Table 1:** Curriculum modules and session topics

Curriculum module	Session topics
Challenging behavior (*n* = 32)	1. Identifying the ABCs of behavior 2. Identifying the function of behavior 3. Preventative and reactive procedures 4. Replacement behaviors 5. Behavior change: generalization and maintenance 6. Behavior change: generalization and maintenance
Communication (*n* = 61)	1. Understanding the ABCs of behavior and learning 2. Identifying how and why your child communicates 3. Using the framework for communication 4. Applying the framework for teaching communication 5. Applying the framework for teaching communication 6. Applying the framework for teaching communication
Sleep (*n* = 10)	1. Sleep and ASD 2. Daytime and evening habits to promote successful sleep 3. Follow-up on daytime and evening habits 4. Responding to challenges during the night 5. Follow-up on challenges 6. Generalizing sleep and behavioral strategies
Social play (*n* = 4)	1. Understanding ABCs of behavior and learning 2. Sensory social routines and communication during play 3. Following the lead and structuring joint activity routines 4. Imitation and turn-taking 5. Sharing interest 6. Independent play
Toilet training (*n* = 2)	1. Child and caregiver readiness—data collection 2. Set goals and develop a schedule 3. Identify issues, additional supports, and reinforcement 4. Functions and responding to challenging behavior 5. Promoting independence 6. Moving forward—planning for generalization

The primary goal of each module was to provide caregivers with evidence-based strategies that could be feasibly implemented within daily routines and activities. For each family, a curriculum module was collaboratively selected by the caregiver and consultant prior to the first intervention session based on caregiver-reported concerns. Intervention sessions were conducted in the family’s primary language, either by a consultant that spoke the language (i.e., Spanish) or through the use of a translator. Three participating families received services in a language other than English. Each module was based on the principles of ABA and utilized evidence-based practices for individuals with ASD to address common concerns of caregivers.

Modules were broken into six sessions, with each session building on the previous. Each session was designed to be conducted in 60-90 min. Sessions were scheduled based on child, caregiver, Part C early intervention provider (EIP), and consultant availability, and generally occurred every 1-2 weeks (range 3-93 days). Instances where sessions occurred more than 2 weeks apart were at the request of the family (e.g., scheduling conflicts; extended vacations; pauses due to the birth of a new baby). Session tools provided in each module included lesson plans, topic-focused tip sheets, and individual planning and practice activities. Each module was available in print and via web-based interactive courses. Consultant guides were also available to accompany each module.

Eligible families who opted to receive behavioral intervention and support services after the diagnostic evaluation first participated in a phone interview with a behavioral consultant. This interview served to share information about the intervention program, answer families’ initial questions, and select a primary intervention goal. During the phone interview, behavioral consultants asked caregivers questions about their child’s development, history with intervention services, caregiver-perceived child strengths, and caregiver-perceived child challenges. Behavioral consultants also provided caregivers with curriculum module options for intervention (e.g., communication, challenging behavior, and social play) and asked caregivers to choose one priority area based on information shared during the interview. If caregivers expressed interest in more than one module, behavioral consultants helped caregivers to select an initial curriculum and offered to share additional resources from other modules during later sessions.

During the first intervention session, consultants offered to review the psychological evaluation report and diagnosis with the family, if applicable and requested. Consultants also provided an overview and introduction to the curriculum module agreed upon during the phone interview. The remainder of this first session and subsequent sessions included didactic instruction on specific teaching strategies, individualization of the strategies to the caregiver and child, modeling, and guided practice and feedback. Tip sheets, video models, and planning guides were used as needed. At the end of each session, caregivers and consultants identified specific activities or skills to practice prior to the next visit. The family’s EIP was invited and encouraged to attend at least two of the six visits to facilitate collaboration and continued support at the end of services. The two sessions they chose to attend were based solely on their availability. Among families who received in-person services, EIPs attended at least two visits for most families (80%; see Table 2).

**Table 2:** Participant demographics

	Full sample *n* (%)	In-person only *n* (%)	Telemedicine-only *n* (%)	Hybrid *n* (%)
Toddlers				
*N*	115	49	46	20
Age in months (*m* [*SD*])	27.97 (4.69)	27.96 (4.67)	28.17 (4.58)	27.50 5.18
Male	85 (74%)	36 (74%)	33 (72%)	16 (80%)
Female	24 (21%)	11 (22%)	10 (22%)	3 (15%)
Opted not to provide	6 (5%)	2 (4%)	3 (7%)	1 (5%)
Toddler race				
White	75 (65%)	36 (74%)	25 (54%)	14 (70%)
Black or African American	11 (10%)	3 (6%)	5 (11%)	3 (15%)
Asian	4 (4%)	1 (2%)	2 (4%)	1 (5%)
Multi-racial	10%	4 (8%)	6 (13%)	2 (10%)
Other	7 (6%)	2 (4%)	6 (9%)	-
Toddler ethnicity				
Hispanic or Latino	11 (10%)	5 (10%)	5 (11%)	1 (5%)
Not Hispanic or Latino	98 (85%)	41 (84%)	38 (83%)	20 (95%)
Opted not to provide	6 (5%)	3 (6%)	3 (7%)	-
Toddler diagnosis status				
Autism spectrum disorder	90 (78%)	41 (84%)	32 (70%)	17 (85%)
Developmental delay	15 (13%)	5 (10%)	8 (17%)	2 (10%)
Other	10 (9%)	3 (6%)	6 (13%)	1 (5%)
Family annual income				
Less than $25,000	21 (18%)	12 (25%)	6 (13%)	3 (15%)
$25-50,000	30 (26%)	9 (18%)	13 (28%)	8 (40%)
$50-75,000	14 (12%)	5 (10%)	7 (15%)	2 (10%)
$75-100,000	14 (12%)	7 (14%)	5 (11%)	2 (10%)
Over $100,000	16 (14%)	7 (14%)	7 (15%)	2 (10%)
Not reported	20 (17%)	9 (18%)	8 (17%)	3 (15%)
Distance from clinic (miles; mean [range])	59.72 (7.1-196)	67.47 (7.1-196)	51.40 (7.5-162)	59.37 (7.4-171)
Intervention sessions received (*m* [*SD*])	5.84 (0.45)	5.80 (0.54)	5.83 (0.44)	6.00 (0)
Intervention sessions attended by EIP (*m* [*SD*])	3.47 (1.90)	3.18 (1.74)	3.87 (2.05)	3.25 (1.83)

Following the shift to telemedicine-only services, families received six, 60-90-min intervention sessions provided via telemedicine. To continue to engage the families’ Part C providers, EIPs were invited to sessions and sent video conferencing links to facilitate participation. EIPs attended at least two visits for most families who received telemedicine-only services (78%; see Table 2). Overlapping sessions by EIPs ranged from 0-6, with the telemedicine group having the highest percentage of EIPs attending all six sessions (in-person, 10%; telemedicine, 24%; hybrid, 13%). There were no significant differences in number of sessions attended by EIPs across groups.

Consultants spent time prior to the first session ensuring that caregivers were able to access and utilize the video conferencing platform. Session format and content were similar to procedures described above, with some modifications necessary given the telemedicine platform, (e.g., use of video models instead of the consultant modeling a strategy for a family).

### Interventionists

Behavioral intervention and support services were provided by 14 consultants with expertise in ASD and behavior analysis. Consultants included board certified behavior analysts (BCBAs), speech-language pathologists, and early childhood educators. Consultants had an average of 11 years of experience working with families and young children with ASD (range = 3 to 25 years) and were supervised by a BCBA-D and two additional BCBAs. Training and supervision of consultants included direct observation and consultation with supervisors and participation in ongoing professional development activities related to ASD and EI (i.e., attendance at local conferences and meetings).

### Participating families

Data for the present study are drawn from families eligible for behavioral intervention and support services between July 2019 and August 2020. A total of 165 families were eligible for participation during this period. Twenty-two families declined participation, including three families who specifically declined telemedicine services. Of the 143 families who expressed interest and initiated program participation, 11 families discontinued or were lost to follow-up before providing demographic or pre-intervention data. Ten families discontinued participation after completing initial sessions (e.g., moved out of state prior to finishing services, family schedule changed), and seven families completed the intervention program but did not provide post-intervention data. Table 2 provides information about the average number of sessions completed by families in each group.

Data analyzed for the present study are drawn from 115 families who completed behavioral intervention and support services, including having available data for at least one post-intervention measure. Forty-nine families participated in in-person only services between July 2019 and March 2020. Forty-six families participated in telemedicine-only services between March 2020 and August 2020. An additional 20 families received hybrid services consisting of some in-person and some telemedicine appointments. All families had a participating child between the ages of 16 and 33 months (*m* = 27.97 months, *SD* = 4.69 months). Participating caregivers were primarily biological parents (86%). Other caregivers included foster parents (*n* = 1), adoptive parents (*n* = 4), grandparents (*n* = 4), and other guardians (*n* = 2). Additional demographic data are presented in Table 2. Families included in the present analyses did not differ from the 17 families who discontinued participation or did not provide any post-intervention data in terms of child gender (*X*^*2*^ = 2.33, *p* > .05), child age (*t* = 0.43, *p* > .05), child race (*X*^*2*^ = 0.67, *p* > .05) or ethnicity (*X*^*2*^ = 1.77, *p* > .05), child diagnostic status (*X*^*2*^ = 1.68, *p* > .05), or distance the family lived from clinic (*t* = 0.43, *p* > .05).

### Measures

We assessed several aspects of these model service programs, including feasibility of implementation, consultant and caregiver satisfaction, and clinical impact of services. To assess clinical impact of services, we gathered consultant and caregiver ratings of child symptom improvement, as well as pre- and post-intervention measures of communication skills. We evaluated Part C system acceptance through the administration of satisfaction surveys to EIPs.

### Feasibility of implementation

#### Completion rates

Participant attendance was tracked by consultants and supervisory staff in a local and system level database. Participants had the opportunity to receive up to six intervention sessions.

#### Treatment fidelity

Treatment fidelity was assessed using a checklist outlining skills introduced in each curriculum module. Generally, each session included three to five objectives. Objectives were related to specific strategies and caregiver implementation of strategies with their child (e.g., “identify how your child is communicating,” “identify next steps for communication”). At the end of each visit, the consultants assessed the number of objectives from the corresponding session number that they were able to discuss with the caregiver. They also assessed the number of skills from the corresponding session that the caregiver was able to demonstrate during the session. Each item was scored as “yes” (this item was discussed/achieved), or “no” (this item was not discussed/achieved). In situations in which a consultant did not have enough time to discuss a curriculum item or determined that the caregiver needed to spend additional time on other items, as opposed to introducing additional strategies, a score of “no” would be given for both consultant discussion and caregiver achievement. At the end of services, the consultant completed the checklist again to determine which objectives had been maintained.

### Impact of services

#### Clinical global impressions of improvement (CGI-I)

Caregivers and consultants completed a clinical global impressions of improvement (CGI-I) scale at the completion of services. These ratings are on a 7-point Likert scale, with higher scores indicating worsened functioning and lower scores indicating improved functioning. The CGI-I rating assesses improvement across the following domains: child participation in caregiving routines, participation in play-based routines, verbal communication, nonverbal communication, social interactions, restricted or narrow interests, and challenging behavior.

#### MacArthur-Bates Communicative Development Inventory (MCDI)—short form [24]

The MCDI is a parent-report instrument that captures information on a child’s developing abilities in early language, including vocabulary comprehension and production. Caregivers were asked to complete the MCDI-short form prior to and following intervention.

#### Communication and Symbolic Behavior Scale - Developmental Profile (CSBS DP) [25]

The CSBS DP measures seven language predictors including emotion and eye gaze, communication, gestures, sounds, words, understanding, and object use. Caregivers were asked to complete the CSBS DP at prior to and following intervention.

#### Caregiver, EIP, and consultant satisfaction surveys

A 14-item questionnaire developed by our internal team assessed caregiver and EIP satisfaction with the service model and consultant, as well as perceived impact on self and child (e.g., “The consultant was knowledgeable about interventions”). Respondents rated 12 items on a scale of 1 to 4, with “1” representing “strongly disagree” and “4” representing “strongly agree.” Caregivers and EIPs were also asked to respond to two open-ended questions regarding aspects of the service they found to be most helpful and recommendations for improvement.

A separate survey regarding caregiver satisfaction with telemedicine services was sent to caregivers who completed at least one telemedicine session. Finally, consultants completed a survey designed to assess perceptions of and comfort with telemedicine-delivered intervention procedures, as well as perceived benefits and challenges associated with this method of service delivery.

### Data collection procedures

When a family verbally accepted services, a service team member contacted the family’s early intervention service coordinator to notify him/her that the service needed to be added to the family’s individualized family service plan (IFSP). Once the service was added to the IFSP, a consultant contacted the family to conduct an initial interview, collaboratively identify service goals for the caregiver and child, and schedule sessions. For in-person services, consultants brought an iPad to the first visit, and the caregiver completed the pre-intervention measures using a secure data collection application prior to the start of the visit. Post-intervention measures were completed in the same manner at the end of the final visit. If the caregiver was unable to complete the measures via iPad for any reason, then a paper copy was provided and entered into the database by a consultant. For telemedicine-delivered services, caregivers were sent a link to an electronic version of the pre-intervention forms prior to the first session. A link to the post-intervention forms was sent at the conclusion of services.

### Analytic plan

We analyzed consultant- and caregiver-reported intervention outcomes, as well as consultant, caregiver, and EIP satisfaction, across three models of service delivery. Data from families who received in-person behavioral intervention and support services between July 2019 and March 2020 (*n* = 49) are compared with data from families who received telemedicine-only intervention services between March 2020 and August 2020 (*n* = 46). A third group is represented by families who received a hybrid model of some in-person and some telemedicine-based services (*n* = 20) between July 2019 and June 2020 (see Table 2). Due to missing data from some families, sample size differs across measures. Unless specified, Kruskal-Wallis *H* tests were used to compare outcomes across groups due to the ordinal and scale nature of the variables being tested; the small sample size of the hybrid group compared to “telemedicine-only” and “in-person” groups; and lack of normality and presence of outliers identified through visualization of data box plots.

## Results

### Feasibility of implementation

Across groups, families completed between three and six intervention sessions (*m* = 5.84, *SD* = 0.45). The total number of sessions completed by families was not significantly different across groups (i.e., in-person only vs. telemedicine-only vs. hybrid; Kruskal-Willis *H* (2) = 3.62; *p* > .05). Most families completed all six sessions of the intervention model (i.e., in person, 78%; telemedicine, 80%; hybrid, 90%). Nearly all families across each group completed at least five intervention sessions (i.e., in-person, 93%; telemedicine, 93%; hybrid, 99%).

A one-way MANCOVA analysis was used to examine consultant-reported treatment fidelity across groups. Average treatment fidelity did not differ significantly across groups (Wilks’ Lambda = 0.942, *F* (4, 194) = 1.469, *p* = .21). Overall, consultants reported completing an average of 82% of treatment objectives during the course of the intervention. Consultants reported that, on average, 70% of objectives were maintained over the course of the intervention.

### Impact of services

Analysis of CGI-I data indicated that most caregivers and consultants reported improvements in child functioning following intervention (see Table 3). Differences across groups emerged for caregiver report of improvement in caregiving routines (Kruskal-Wallis *H* (2) = 13.19; *p* < .05) and social interactions (Kruskal-Wallis *H* (2) = 6.85; *p* < .05). Caregivers who received in-person services reported more improvement in caregiving routines and social interactions (median rank for both = 2.00 indicating “much improved”) than did caregivers who received telemedicine-only services (median rank = 3.00 [“minimally improved”] for caregiving routines and 2.00 for social interactions). Differences across groups also emerged for consultant report of improvement in the areas of play (Kruskal-Wallis *H* (2) = 6.77; *p* < .05), nonverbal communication (Kruskal-Wallis *H* (2) = 7.11; *p* < .05), and social interactions (Kruskal-Wallis *H* (2) = 6.61.; *p* < .05). Across each of these domains, consultants for families receiving in-person services reported more improvement (median rank = 2.00 indicating “much improved”) than did consultants for families receiving telemedicine-only services (median rank = 3.00; “minimally improved”).

**Table 3:** Consultant and caregiver-reported functional improvement

Functional domain	Caregiving routines	Play	Verbal communication	Nonverbal communication	Social behavior	Restricted, repetitive behavior	Challenging behavior
Very much improved (%)							
Consultant	10	10	10	10	11	0	10
Caregiver	10	19	12	19	17	8	12
Much improved (%)							
Consultant	44	46	28	38	43	17	31
Caregiver	42	49	31	43	45	29	31
Minimally improved (%)							
Consultant	44	39	48	45	39	50	35
Caregiver	35	29	37	29	29	37	39
No change (%)							
Consultant	2	4	14	6	6	32	21
Caregiver	13	4	20	8	8	25	12
Minimally worse (%)							
Consultant	1	0	0	0	0	1	2
Caregiver	1	0	0	0	0	0	4
Much or very much worse (%)							
Consultant	0	0	0	0	0	0	0
Caregiver	0	0	0	0	0	0	1

Caregiver data was available for 84 families. Consultant data was available for 114 families

Across all groups, caregivers reported specific improvements in child communication development, as measured by the MCDI and CSBS DP. Paired-sample *t* tests were used to compare caregiver ratings prior to and following intervention. Overall, on the MCDI, caregivers reported that their children were able to say and understand more words following intervention (*m* = 17.40, *SD* = 21.52) than prior to intervention (*m* = 11.05, *SD* = 16.44; *t* (72) = −5.43; *p* < .05). There were no significant differences across groups (in-person only vs. telemedicine-only vs. hybrid) regarding change in caregiver ratings of their child’s ability to say (Kruskal-Wallis *H* (2) = 5.860, *p >* .05) or say and understand words (Kruskal-Wallis *H* (2) = 1.311, *p >* .05).

Similarly, caregivers reported increases across all composite scores on the CSBS DP from pre- to post-intervention (see Table 4). This indicates that caregivers noticed increased use of nonverbal social communication strategies (e.g., eye gaze, gestures), vocalizations (e.g., sounds, words), and symbolic communication (e.g., object use). There were no significant differences across groups in change from pre- to post-intervention on the CSBS DP (i.e., *p* > .05 for all Kruskal-Wallis tests).

**Table 4:** Communication and symbolic behavior scale (CSBS DP) composite scores pre- and post-intervention

	Pre	Post	*t*	*df*	*p*
Social-weighted raw score	26.72 (10.92)	30.27 (8.64)	3.45	70	< .01
Speech-weighted raw score	15.34 (9.68)	18.24 (10.44)	3.53	69	< .01
Symbolic-weighted raw score	22.76 (11.41)	27.00 (12.08)	3.88	69	< .01

### Caregiver satisfaction

Seventy-two caregivers completed satisfaction surveys following intervention (see Table 5). The majority of caregivers reported that the consultant with whom they worked was knowledgeable about interventions (92% reported “strongly agree”), communicated clearly (90% reported “strongly agree”), and provided useful recommendations (92% reported “strongly agree”). Caregivers also reported feeling satisfied with the outcomes of services (86% reported “strongly agree”). No significant differences in caregiver satisfaction emerged across groups.

**Table 5:** Caregiver satisfaction with intervention services

	Strongly agree (%)			Agree (%)			Disagree or strongly disagree (%)		
	IP^a^	TM^a^	Hyb.	IP^a^	TM^a^	Hyb.	IP^a^	TM^a^	Hyb.
The objectives of the consultation were clear.	83	82	87	17	19	13	0	0	0
Appointments and home visits were appropriate in length and scheduled at convenient times.	90	93	87	10	7	13	0	0	0
The consultant was knowledgeable about interventions.	90	96	87	10	0	13	0	4	0
The consultant was knowledgeable about child development and my child’s developmental challenges.	90	96	73	10	0	27	0	4	0
The consultant understood and addressed our needs.	90	89	87	10	7	13	0	4	0
The consultant was well prepared and well organized.	93	93	87	7	7	13	0	0	0
The consultant communicated clearly.	90	93	87	10	7	13	0	0	0
The consultant provided useful recommendations.	93	93	87	7	7	13	0	0	0
My child’s behavior and skills improved during this service.	77	78	60	23	19	40	0	4	0
The final report provided was understandable and useful.	87	78	80	10	22	20	0	0	0
I was pleased with the outcome of services for me and my child.	87	85	87	10	11	13	0	4	0
I would recommend these services to other families.	87	89	87	10	7	13	0	4	0

^a^Data available for 72 caregivers. *N* = 30 for in-person (IP) group; *N* = 22 for telemedicine-only (TM) group; *N* = 15 for hybrid group

Caregivers who received telemedicine services were asked to provide specific feedback about their satisfaction with the telemedicine modality. A total of 28 caregivers in the telemedicine-only group completed this survey. The majority of caregivers reported feeling that the telemedicine consultant was engaged during the session (86% endorsed “strongly agree”), that they were able to communicate their concerns to the consultant (89% endorsed “strongly agree”), and that the telemedicine session was private (75% endorsed “strongly agree”). Qualitatively, caregivers commented that telemedicine visits were convenient (*n* = 7) and provided an opportunity to continue services during the COVID-19 pandemic (*n* = 2).

### EIP satisfaction

A total of 40 EIPs completed satisfaction surveys regarding their participation in behavioral intervention and support services. Like caregivers, the majority of EIPs reported that consultants were knowledgeable about interventions (83% endorsed “strongly agree”) and communicated clearly (85% endorsed “strongly agree”), and that they would recommend participation in the intervention model to other families (90% endorsed “strongly agree”). Statistically significant differences emerged between EIPs who participated in the hybrid service model and those who participated in the telemedicine-only model on items related to consultant knowledge about interventions (Kruskal-Wallis (2) = 6.37; *p* <.05) and child development (Kruskal-Wallis (2) = 6.65; *p* <.05), consultant preparation (Kruskal-Wallis (2) = 6.65; *p* <.05) and communication, (Kruskal-Wallis (2) = 7.84; *p* <.05), and overall satisfaction with service outcomes (Kruskal-Wallis (2) = 6.70; *p* <.05). For all items with significant differences, EIPs who participated in telemedicine-only services provided higher ratings (median rating = 4.00) than did EIPs who participated in the hybrid model (median rating = 3.50).

### Consultant satisfaction with telemedicine services

Consultants reported feeling comfortable with all aspects of telemedicine-delivered services, including establishing rapport with families, conducting caregiver observations, monitoring progress, and providing feedback and recommendations to families (see Table 6). All consultants reported that telemedicine would be an acceptable method of EI service delivery, with the majority of respondents agreeing that this modality would be appropriate for children across a range of impairment (85%) and behavioral concerns (85%). Although consultants acknowledged the problem of limited availability of EI services, only 54% of consultants indicated that it is appropriate for a family to receive services exclusively over telemedicine. The majority of consultants reported planning to continue using telemedicine in their clinical practice after normal operations resume (92%).

**Table 6:** Consultant perceptions of telemedicine

	Agree strongly	Agree somewhat	Neutral	Disagree somewhat	Disagree strongly
Telemedicine would be an acceptable method for providing EI services to toddlers (15-36 months)	8 (62%)	5 (39%)	-	-	-
Telemedicine-delivered EI services would be appropriate for children across a spectrum of impairment (i.e., low vs. high)	3 (23%)	8 (62%)	-	2 (15%)	-
Telemedicine-delivered EI services would be appropriate for children across a range of concerns (e.g., communication, and challenging behavior)	4 (31%)	7 (54%)	1 (8%)	1 (8%)	-
The problem of limited availability of EI services is an important problem and is large enough to justify the use of telemedicine.	11 (85%)	1 (8%)	1 (8%)	-	-
Parents would find telemedicine to be an appropriate method for providing EI services to toddlers.	2 (15%)	9 (69%)	1 (8%)	1 (8%)	-
The use of telemedicine-delivered EI services is unlikely to result in serious negative outcomes for the child.	11 (85%)	2 (15%)	-	-	-
	Appropriate	Neutral	Inappropriate		
How appropriate do you feel it is for a family to receive EI services exclusively over telemedicine?	7 (53.8%)	5 (38.5%)	1 (7.7%)		
	Extremely comfortable	Somewhat comfortable	Not at all comfortable		
How comfortable do you feel providing EI services using telemedicine?	6 (46%)	7 (54%)	-		
How comfortable do you feel establishing rapport with families during a telemedicine visit?	8 (62%)	5 (39%)	-		
How comfortable do you feel conducting observations of caregiver-implemented recommendations during a telemedicine visit?	10 (77%)	3 (23%)	-		
How comfortable do you feel monitoring progress during a telemedicine visit?	7 (54%)	6 (46%)	-		
How comfortable do you feel providing recommendations and feedback to families during a telemedicine visit?	10 (77%)	3 (23%)	-		

Qualitatively, consultants provided feedback on the benefits and challenges of telemedicine-based services. Reported benefits included reductions in travel and transportation barriers for families, as well as additional scheduling flexibility for caregivers with busy schedules (*n* = 5). Some consultants also reported an increase in family engagement during visits, with caregivers taking a more active role in treatment protocols (*n* = 5). Consultants also reported logistical benefits related to telemedicine. For example, consultants were able to take more time to prepare for visits (*n* = 1) and increase their capacity for the number of families they serve in a week (*n* = 5) as they did not need to travel to families’ homes. They also reported that it was easier to schedule sessions and document visits electronically, as well as to observe other consultants in order to provide peer coaching and troubleshoot problems (*n* = 5).

Regarding challenges experienced during telemedicine sessions, 83% of consultants reported experiencing technology-related challenges, including unreliable internet connections and difficulty helping caregivers to set up and adjust the camera throughout the appointment to ensure adequate observations. Some consultants (*n* = 5) reported challenges related to modeling certain behaviors for parents and providing examples over telemedicine. Consultants also reported some initial difficulty establishing caregiver buy-in for telemedicine-delivered services (*n* = 2) and establishing rapport (*n* = 3). Finally, one consultant reported difficulty addressing severe or aggressive behavior over telemedicine.

## Discussion

The present study describes outcomes of a brief behavioral intervention and support program for caregivers of children with developmental concerns, with a specific focus on distinct models of service delivery implemented prior to and during the COVID-19 pandemic. The shift in services from in-person to a telemedicine-based model presented an opportunity to investigate perceptions of and outcomes associated with telemedicine services. The data presented above suggest that a brief behavioral intervention and support service, whether provided in person or via telemedicine, may positively impact caregiver and child behavior. Both caregivers and EIPs reported high levels of satisfaction with services, regardless of delivery method, and caregivers and consultants shared positive feedback regarding telemedicine-only services. They also provided informative feedback regarding perceived barriers to care provision of likely relevance to future work.

Results of this study suggest that caregivers perceive even a brief behavior-focused intervention as beneficial. In addition to feeling satisfied with services, both caregivers and consultants reported improvements in child outcomes following intervention. Caregivers reported specific improvements in child communication over the course of intervention, regardless of the method of intervention delivery. These positive outcomes following a brief intervention may provide support for strained systems of care to implement at least brief intervention protocols when more intensive services are not widely available.

In addition to caregivers and consultants, EIPs who attended intervention sessions reported feeling satisfied with brief intervention services. Although follow-up data from EIPs were available for only a portion of involved providers, those who did respond identified several benefits of engaging in collaborative treatment sessions with consultants. This represents perhaps one of the most important and meaningful impacts of embedded community intervention work, beyond the immediate benefits as felt by families themselves—that is, the ripple effect of partnerships between Part C providers and our consultants across other children served by the Part C system. Building and promoting these partnerships to allow systems to work together in a streamlined way not only enhances the ability of both to provide care but also provides embedded training opportunities to further disseminate evidence-based practices [14, 15].

The results of this study suggest that telemedicine is a viable strategy for overcoming the geographic barriers many families face in accessing early behavioral intervention services. Often, access to specialized early intervention services such as ABA, speech-language therapy, and occupational therapy can be limited for families living in rural or under-resourced areas [9, 10]. One challenge families face is identifying a provider able to travel to communities far from the provider’s clinic or own home. It may be equally challenging for families to travel to specialty clinics to receive services. A family’s ability to travel to these appointments may be limited by the flexibility of their jobs, cost of fuel, and general availability of transportation. Telemedicine offers families the opportunity to connect to experts from their own homes, without barriers associated with travel to a distant clinic [10].

For providers, telemedicine allows for observation and treatment of caregivers and children in their natural environment, an important component of quality early intervention services [26], without having to travel a great distance to do so. As the number of children in need of early intervention services continues to rise, so does the demand for early intervention providers to carry large caseloads. However, for those providers who typically see families in homes, the number of families they can see in each day is limited by the distance required to travel between appointments. Eliminating travel through use of telemedicine models of service delivery may subsequently increase the time providers have to serve additional families. Furthermore, eliminating travel reduces costs to the provider and/or the agency who may be responsible for providing mileage reimbursement [27].

Across the majority of outcomes measured, there were few statistically significant differences between families receiving in-person only, telemedicine-only, or hybrid service models. These promising results support the use and value of telemedicine services. However, some differences did emerge between caregivers and consultants participating in in-person versus telemedicine-only interventions, with both caregivers and consultants reporting relatively less perceived improvement in child outcomes following telemedicine-only services. Though improvements were still reported, these were less in magnitude for those engaged in telemedicine-only services. Given this, additional research is needed to examine both objective and subjective measures of child and family outcomes following telemedicine intervention.

Consultants also reported several unique considerations when converting in-person services to telemedicine. The ability to coach caregivers from a distance is critical to working with families via telemedicine. While many of the same components of effective coaching apply across both models of service delivery, some strategies (i.e., modeling) necessarily look different across the two models. Additionally, telemedicine is still a novel model of service delivery for many families. As noted in our results, some consultants found that it took time to establish rapport and secure caregiver buy-in via telemedicine. As telemedicine continues to grow as a model of service delivery across behavioral health fields, attention should be given to which components of practice translate most cleanly, which require modification, what effective modification looks like, and importantly—which components of practice do not cleanly or safely translate. Training and fidelity measures on service provision via telemedicine will be essential to quality control.

### Limitations and future directions

While the promise of telemedicine is great, it is not without limitation. Implementation of services via telemedicine requires that both parties—the provider and the consumer—have a reliable device that is compatible with video conferencing software. For providers, additional consideration must be given to HIPAA and FERPA guidelines when selecting a telemedicine platform. Reliable internet access can be another barrier to successful implementation of telemedicine. Despite more widely available broadband services, there are still communities and neighborhoods in which a consistent cellular signal is challenging to obtain. Poor connections impact audio and video quality, which directly impact the quality of an intervention. Even when cellular service is adequate, there are many families who cannot afford a monthly internet charge and have zero or very limited access to wireless internet connections.

Future efforts should focus not only on strategies for overcoming challenges related to poor internet connectivity but also on how to serve families without internet access. For example, developing strategies for sharing technology (e.g., internet hot spots, devices equipped with telemedicine platforms) or providing EIPs with such technology for use in families’ homes may present opportunities for increasing service access. Prior tele-assessment work has demonstrated the value of satellite clinics at which families can connect to providers in distant locations [19]. Similarly, designating clinics or community centers in under-resourced neighborhoods as sites for the delivery of telemedicine interventions may help to overcome technology-related barriers.

Previous work has demonstrated that some families in rural and under-resourced communities are not referred for services at all due to the barriers described above [19]. It is likely that there are many more families in need of services within these communities than we currently understand. Future study should focus on how to (1) identify these families, (2) understand the specific needs of these families and their communities, and (3) build sustainable systems for reaching these families. The success of the model described above can be attributed to our partnership with the Part C system and efforts to prioritize shared areas of need. We built this model by listening to the concerns of the providers already in these communities and engaging them in both the development and implementation of each component. Building this model within an existing, large-scale system for young children has helped not only in sustaining the model but also in its ability to be replicated and grown over time.

Finally, it is vital to continue investigating facilitators and barriers of the success of telemedicine-based services, including understanding for whom telemedicine works best. In the present study, three families declined telemedicine services. Additional families discontinued both in-person and telemedicine services. Though these families did not differ from families who completed services along any of the demographic factors analyzed, it is possible that these families differed in other ways. Understanding how best to serve and retain families throughout the course of intervention, regardless of the modality of intervention delivery, is an important aspect of service provision and related research. Finally, the current research is limited by its reliance on outcome measures completed by stakeholders (i.e., caregivers and intervention providers). Although these perspectives are vital for assessing the feasibility and sustainability of novel service delivery models, there is a need for research utilizing validated and objective measures of child improvement completed by external raters.

## Conclusions

The present work has provided an opportunity to examine and compare telemedicine and in-person service delivery models within the context of the shift in services necessitated by the COVID-19 pandemic. These results provide preliminary support for the feasibility of delivering behavioral intervention and support services via telemedicine and in partnership with community providers. This type of caregiver education and brief behavioral intervention—whether delivered in person or via telemedicine—has demonstrable positive impacts on caregiver behavior and perceptions of child behavior. Ultimately, embedding opportunities for telemedicine-based services may allow a larger number of families to experience these benefits.

## Data Availability

Deidentified datasets and other materials (e.g., questionnaires, curriculum modules) described in this publication are available from the corresponding author on reasonable request.

## Abbreviations

ASD: Autism spectrum disorder
ABA: Applied behavior analysis
EIP: Early intervention provider
EI: Early intervention
BCBA: Board certified behavior analyst
MCDI: MacArthur-Bates Communicative Development Inventory
CSBS DP: Communication and Symbolic Behavior Scale—Developmental Profile
